# Fast graph convolutional models incorporating matrix factorization for predicting microbe-disease associations

**DOI:** 10.1038/s41598-025-30284-y

**Published:** 2025-12-10

**Authors:** Qingwen Wu, Sujuan Tang

**Affiliations:** 1 Department of Data Center, Affiliated Hospital of Jining Medical University, Jining, China; 2 Department of Neurological Care Unit, Affiliated Hospital of Jining Medical University, Jining, China

**Keywords:** Microbe-disease association, Matrix factorization, Graph embedding, Spatial convolution, Machine learning, Computational biology and bioinformatics, Mathematics and computing

## Abstract

Revealing the relationship between microbe and disease is of great significance to the diagnosis, treatment, and prevention of disease. To overcome the expensive cost and trial-and-error settings, a series of in-silico methods have been proposed to predict microbe-disease association. However, the predictive performance of the current methods is modest. In this paper, we propose a new computational method based on Fast Graph Convolutional and Matrix Factorization, called FGCNMF, which addresses microbe-disease association prediction as a binary classification task by learning embedding representation of nodes on a microbe-disease network. We integrate background information from both microbe and disease spaces into the same global network framework, and use the randomized Singular Value Decomposition algorithm to obtain high-quality initial embedding representations of node. Then, Fast Spatial Convolution is implemented to enhance the embedding representations. Finally, using the enhanced representation of node pairs as input, and using Extra-Trees classifier to predict the final label. Experimental results demonstrate that FGCNMF has improved performance in comparison with other state-of-the-art computational methods on the benchmark datasets.

## Introduction

An intricate, co-evolutionary relationship exists between humans and a vast consortium of resident microorganisms, primarily bacteria. These microbes colonize nearly every part of the human body, with particularly dense populations found in the gastrointestinal tract and on the skin^1^, forming complex ecosystems critical to host physiology.

The skin’s microbial community, for instance, serves as a protective barrier. It is largely composed of beneficial commensals that actively inhibit pathogenic invasion while also playing a crucial role in calibrating the host’s immune responses^2^. This homeostatic balance, however, can be disturbed by a variety of endogenous and exogenous pressures, leading to pathological states^3^. Clinical examples include the association of Staphylococcus aureus with atopic dermatitis and Streptococcus pyogenes with guttate psoriasis^2^.

In the gastrointestinal tract, the microbiota’s influence on human health is even more profound. These microbes are essential for metabolic functions, such as converting dietary fiber into short-chain fatty acids, and for the proper development and functioning of the immune system^4^. Consequently, a disruption of this gut microbial community, a condition known as dysbiosis, is linked to immunological dysfunction and disease pathogenesis. Such disturbances are known to exacerbate risk factors for a range of cardiometabolic disorders, including diabetes, obesity, and atherosclerosis^5^. Specific microbial shifts, such as the depletion of the genus Odoribacter, have been identified as potential biomarkers for distinct pathologies like inflammatory bowel disease (IBD)^6^ and non-alcoholic fatty liver disease.

Unlocking the intricate roles of the human microbiome is paramount for advancing human health, offering pathways to better diagnostics, treatments, and preventative strategies^7^. However, progress in this area is hampered by the current scarcity of validated microbe-disease associations. Traditional discovery methods, which rely on direct biological and clinical experimentation, are inherently slow, costly, and labor-intensive, creating a significant bottleneck in research. To circumvent these challenges, the field has witnessed a surge in the development of computational, or in-silico, methods. These approaches provide a powerful and efficient framework for predicting novel microbe-disease links, and they can be broadly classified into two dominant paradigms^8^: models based on network algorithms and those that employ machine learning techniques.

The first major category, network-based models^9–12^, operates by representing microbes, diseases, and their known relationships as nodes and edges within a complex heterogeneous network. The core task is then to infer missing edges, representing undiscovered associations, by analyzing the network’s topological properties. A variety of strategies have been proposed to tackle this. Some approaches, such as KATZHMDA^13^, quantify the likelihood of an association by calculating the similarity between nodes based on the number and length of paths connecting them. Other methods leverage techniques like random walks (e.g., NTSHMDA^14^ or network consistency projection^15^ (e.g., NCPHMDA^9^, HMDA-Pred^16^ to propagate information across the network and identify promising candidate associations. Despite their ingenuity, a fundamental limitation plagues many of these models: their strong reliance on a pre-existing network of known interactions. Consequently, their predictive performance is often compromised when dealing with new diseases or microbes that have no previously documented links, rendering them ineffective for “orphan” node predictions.

The second prominent strategy leverages machine learning^17–19^, typically framing the prediction task as a binary classification problem. These models excel at learning from complex data by extracting or constructing discriminative features for microbe-disease pairs. A diverse range of architectures has been explored. For instance, deep learning approaches, such as the three-layer neural network in BPNNHMDA^20^ or the deep matrix factorization model in DMFMDA^21^, aim to learn low-dimensional, dense vector representations (embeddings) directly from the data. Other methods, like RNMFMDA^22^, employ sophisticated matrix factorization techniques combined with clever sampling strategies, such as positive-unlabeled learning, to address the common challenge of lacking confirmed negative examples. While these methods have certainly advanced the field, they often face their own hurdles, including challenges in fully capturing the complex topological characteristics of the underlying biological network, which can limit their predictive accuracy and efficiency. To overcome these limitations, more recent research has embraced advanced Graph Convolutional networks (GCN) architectures. Models like GraphSAGE have introduced sophisticated inductive learning capabilities, while Graph Transformers have adapted the highly successful attention mechanism for graph data, enabling the capture of complex global dependencies. On the frontier, sophisticated frameworks combining contrastive learning with graph diffusion are being explored^23^, aiming to enhance representation robustness, though often at the cost of increased model complexity and computational overhead.

To address the aforementioned limitations, this paper introduces FGCNMF, a novel and highly efficient computational framework that synergistically combines Fast Graph Convolutional networks with Matrix Factorization. The novelty of our approach lies in its unique two-stage embedding pipeline that decouples global structure learning from local neighborhood refinement, leading to significant gains in both speed and accuracy. Our key contributions are threefold:

1. An efficient, non-iterative embedding initialization. We bypass traditional end-to-end GCN training by first using Randomized Singular Value Decomposition (SVD) to rapidly generate a high-quality global embedding for each node.
2. A lightweight, parameter-free refinement stage. We then enhance these initial embeddings using simple, fast spatial graph convolutions to effectively integrate local neighborhood information without the computational overhead of complex aggregators or attention mechanisms.
3. A superior balance of performance and efficiency. The resulting framework is not only an order of magnitude faster than contemporary GCN baselines but also achieves state-of-the-art predictive performance on benchmark datasets.

The FGCNMF workflow unfolds in three key stages: First, the initial global node embeddings are generated via Randomized SVD. Second, these embeddings are enhanced through spatial convolutions. Finally, the refined embeddings are used as features for a downstream classifier to predict microbe-disease associations. Through rigorous cross-validation on benchmark datasets and compelling case studies, we demonstrate that FGCNMF achieves state-of-the-art performance, significantly outperforming existing models and establishing it as a powerful and practical tool for accelerating microbiome research.

## Materials and methods

### Dataset

The datasets used in this study were downloaded from previous work^24^. We obtained a dataset HM with 450 known microbe-disease associations and a dataset DB with 4351 known microbe-disease associations (see Table 1). These known microbe-disease associations come from HMDAD^25^ database and Disbiome database^26^, respectively. Furthermore, microbe functional similarity was calculated by the Kamneva method^27^, and disease functional similarity was calculated based on the functional associations between disease-related genes. For convenience, we denoted the known microbe-disease associations as MD, microbe similarity as SM, and disease similarity as SD, respectively.

**Table 1:** The statistical information of microbe-disease datasets.

Dataset	Microbe	Disease	Links	Sparseness
HM	292	39	450	0.0395
DB	1052	218	4351	0.0189

### Microbe-disease network

Similarity information between microbes (or diseases) contains a large amount of topological information. However, a large amount of effective neighbor information is included in the most similar neighbors, and the low-similarity neighbors have little effect on the model. Therefore, for each microbe in *SM*, we find its top-*n* similar neighbors and set edges to connect it with its neighbors. In this way, we obtain a new similarity matrix *SM* by

$$\:SM(i,j)=\left\{\begin{array}{c}\:1,\:\:\:if\:j\in\:top\:n\:neighbors\:of\:i\\\:\:0,\:\:\:\:\:\:\:\:\:\:\:\:others\:\:\:\:\:\:\:\:\:\:\:\:\:\:\:\:\:\:\:\:\:\:\:\:\:\:\:\:\:\:\:\end{array}\right.$$.

Similarly, we can obtain the new similarity matri × *SD* of disease.

Based on the new similarity matrix and known association, we construct microbe-disease network $\:G\:=\:(V,\:E)$. Each node$\:\:i\in\:V$ represents microbe or disease and each edge $\:(i,\:j)\in\:E\:$represents relationship between nodes $\:i$ and $\:j$. The adjacency matrix $\:H$ of $\:G$ is described as follows

$$\:H\:=\left[\begin{array}{cc}SM&\:MD\\\:{MD}^{T}&\:SD\end{array}\right]$$.

where $\:{MD}^{T}$ is the transpose of the matrix $\:MD$. The degree matrix $\:D$ of $\:G$ is a diagonal matrix with $\:{D}_{ii}={\sum\:}_{j}{H}_{ij}$.

### Prediction model FGCNMF

**Fig. 1:**
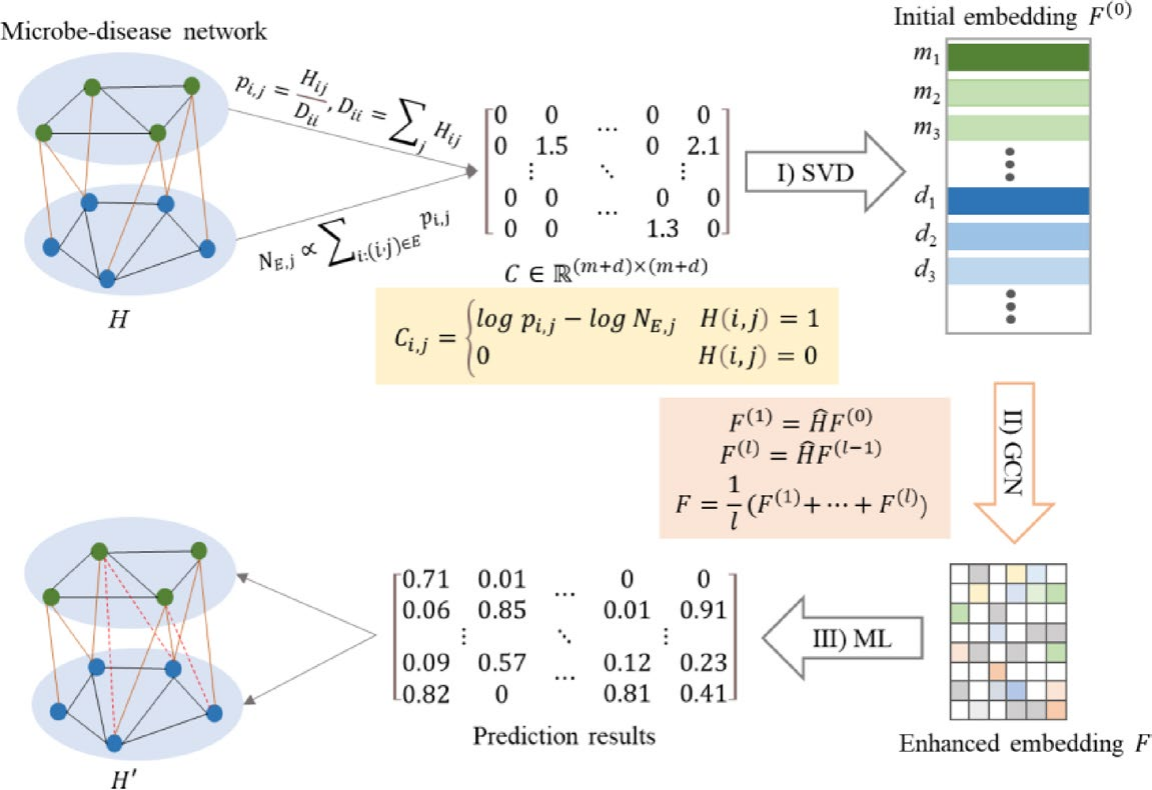
The workflow of FGCNMF.

We propose a novel prediction model FGCNMF which can precisely predict microbe-disease associations. FGCNMF including three steps (Fig. 1): (1) learning the initial embedding vector of graph nodes using Matrix Factorization, (2) enhancing node embeddings via spatial convolution, and (3) using machine learning algorithm to predict associations between unknown node pairs.

Given the network *G*, graph representation learning algorithm will learn a mapping function$\:\:f:\:V\to\:{F}^{d}$ that projects each node to a *d*-dimensional space. It uses a low-dimensional, dense vector to represent the nodes in the graph. This vector represents the structure of the graph, the more adjacent nodes shared by two nodes, the closer the two corresponding vectors are. The biggest advantage of graph representation learning is that the obtained vector representation can be input to any machine learning model to solve specific problems.

Inspired by proNE^28^, we formulates graph embedding as sparse matrix factorization to efficiently achieve initial node representations. Specifically, for node $\:i$ and $\:j$, to representing their similarity in graph *G*, a similarity matrix *C* was defined as

1 $$\:{C}_{i,j}=\left\{\begin{array}{c}log\:{p}_{i,j}-{log}{N}_{E,\:j},\:\:\:\:\:H\left(i,j\right)=1\\\:0,\:\:\:\:\:\:\:\:\:\:\:\:\:\:\:\:\:\:\:\:\:\:\:\:\:\:\:\:\:\:\:\:\:\:\:\:\:H\left(i,j\right)=0\end{array}\right.$$

2 $$\:{p}_{i,j}=\frac{{H}_{ij}}{{D}_{ii}}$$

where $\:{N}_{E,\:j}$ is the negative samples associated with node *j* and defined as

3 $$\:{N}_{E,\:j}\propto\:{\sum\:}_{i:(i,j)\in\:E}{p}_{i,j}$$

Randomized Singular Value Decomposition is applied to achieve fast embedding learning. Assuming

4 $$\:C\approx\:{\text{Q}\text{Q}}^{T}C$$

5 $$\:{Z=Q}^{T}C$$

*Z* can be decomposed efficiently by the standard SVD, that is

6 $$\:Z=S\varSigma\:{V}^{T}$$

Therefore,

7 $$\:C\approx\:Q{Q}^{T}C=QS\varSigma\:{V}^{T}$$

and get the initial node representation

8 $$\:{F}^{\left(0\right)}=QS{\varSigma\:}^{\frac{1}{2}}$$

$\:Q\:$can be obtained by QR decomposition

9 $$\:CU=QR$$

where$\:\:U\sim\mathcal{N}(0,\:1/d)\:$is Gaussian random matrix.

However, embeddings learned by sparse matrix factorization capture only local structural information. To further integrate global structure information of graph, spatial convolution is used to enhance initial embeddings. Inspired by LightGCN^29^, the node representation $\:F$ is updated by

10 $$\:{F}^{\left(1\right)}=\widehat{H}{F}^{\left(0\right)}$$

11 $$\:{F}^{\left(l\right)}=\widehat{H}{F}^{(l-1)}$$

12 $$\:F=\frac{1}{l}{(F}^{\left(1\right)}+\dots\:+{F}^{\left(l\right)}$$

where $\:{\widehat{H}=D}^{-\frac{1}{2}}H{D}^{-\frac{1}{2}}$, *l* represents the number of convolutional layers. The final embedding$\:F$combines the localized smoothing and global clustering information.

Due to the main time cost lies in the SVD of *Z* and the QR decomposition of *C*, it is very efficient to learn the embedding of nodes for microbe-disease networks. Then, we used machine learning algorithm as classifiers to predict potential microbe-disease associations.

## Results

### Experimental settings

To assess the predictive performance of our proposed FGCNMF model, we employed a rigorous 5-fold cross-validation (5-CV) framework. The complete set of known microbe-disease associations was randomly partitioned into five equal-sized subsets. For each fold, the procedure was as follows to ensure a fair evaluation and prevent any data leakage: (1) A single subset of associations was held out as the test set. (2) The remaining four subsets were used as the training set to construct the microbe-disease network. The links from the test set were completely excluded from this network graph. (3) The entire embedding generation process, including both the SVD-based initialization and the spatial graph convolutions, was performed solely on this training graph. (4) The resulting node embeddings were then used to train the final machine learning classifier. (5) Finally, the trained model was evaluated on the held-out test set.

This entire 5-CV procedure was repeated 30 times with different random seeds, and the final results were averaged to mitigate any bias arising from data partitioning. The model’s efficacy was quantified using two standard metrics: the Area Under the Receiver Operating Characteristic Curve (AUC) and the Area Under the Precision-Recall Curve (AUPR).

The task of predicting microbe-disease associations is framed as a binary classification problem, which requires both positive and negative samples for training the classifier and evaluating its performance. The set of all known microbe-disease associations, as described in Section "Dataset", constitute the positive samples in our study. These represent the ground-truth links that the model aims to identify. Identifying confirmed non-associations between microbes and diseases is a significant challenge, as the absence of a recorded link does not definitively prove non-association. Therefore, following common practice in link prediction research, we treat all microbe-disease pairs without a known association in our datasets as unlabeled samples. From this large pool of unlabeled pairs, we construct the negative sample set. To create a balanced dataset for our binary classifier, we randomly select a number of these unlabeled pairs equal to the number of known positive associations. This 1:1 sampling ratio of positive to negative samples is maintained throughout our 5-CV procedure. For each fold, the training set consists of 80% of the positive samples and an equal number of randomly selected negative samples. The remaining 20% of positive samples and an equal number of other unlabeled pairs form the test set, from which performance metrics are calculated. This balanced approach ensures a fair and robust evaluation of the model’s predictive capabilities.

To benchmark FGCNMF’s performance, we conducted a comparative analysis against four state-of-the-art methods. These baselines included: GATMDA^24^, a model utilizing graph attention networks and inductive matrix completion; BRWMDA^30^, which performs a bi-random walk on the microbe and disease similarity networks; GRNMFHMDA^31^, a framework based on graph regularized non-negative matrix factorization for simultaneous prediction across all diseases; and KATZHMDA^13^, a foundational method that predicts associations using the KATZ centrality measure.

### Parameter studies

There are three key parameters in this study, the number of top similar neighbors *n*, the embedding dimension *d*, and the number of convolutional layers *l*. Next, we analyze the impact of different parameter settings on the FGCNMF model. To test the sensitivity of the proposed model to these parameters, we obtained the AUC and AUPR values by varying these parameters at the 5- CV setting.

**Fig. 2:**
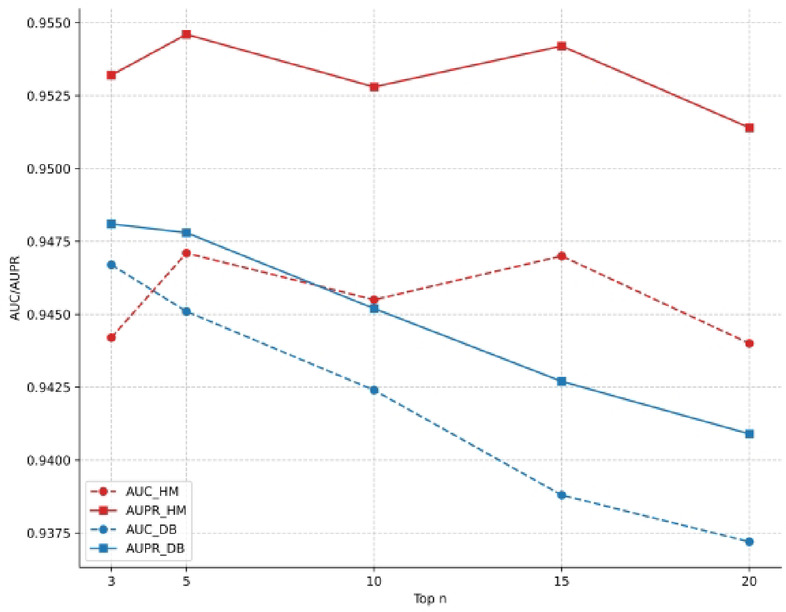
Performance comparison of top n.

The first key hyperparameter in our model is top *n*, which is used in the construction of the microbe-disease network. This parameter controls the sparsity of the similarity networks by filtering out weaker, potentially noisy connections and retaining only the edges to the n most similar neighbors for each node. To investigate the effect of this parameter, we varied n across the values [3, 5, 10, 15, 20] and evaluated the model’s performance. The results are presented in Fig. 2. For the HM dataset, we observe that the model achieves its peak performance for both AUC and AUPR when *n* is set to 5. For the DB dataset, the optimal performance is reached when *n* is 3. The performance on the DB dataset shows a steady decline as *n* increases, suggesting that for this larger and sparser network, including more neighbors beyond the top 3 introduces more noise than useful signal, thereby degrading performance. The HM dataset benefits from a slightly larger neighborhood, but performance also begins to decline as n becomes too large. This highlights the importance of tuning this parameter to strike the right balance between information retention and noise filtering. Based on these findings, we set *n* = 5 for the HM dataset and *n* = 3 for the DB dataset in all other experiments conducted in this study.

**Fig. 3:**
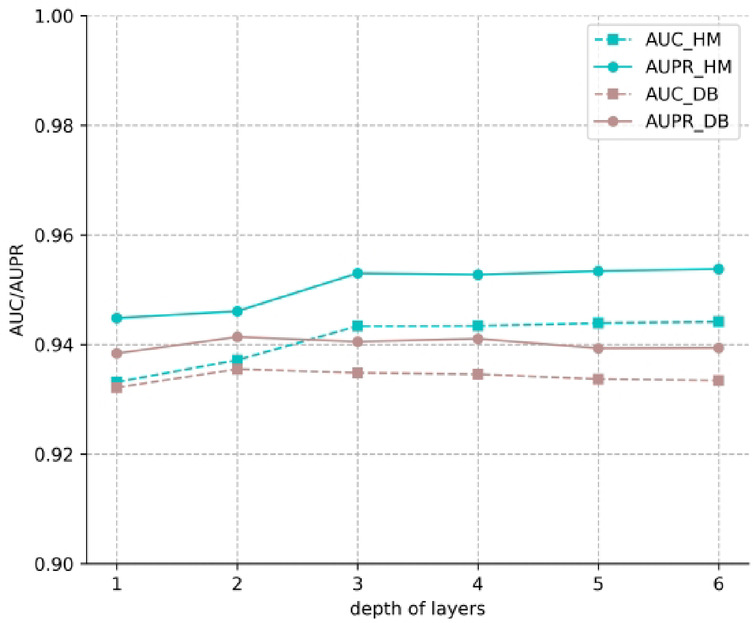
Performance comparison of layer depths in GCN.

Second, we chose$\:\:l\:$from 1, 2, 3, 4, 5, and tested it on dataset HM and DB. Figure 3 describes the changes of average AUC and AUPR scores. We can find that when $\:l$ reaches a certain value, the performance of the model plateaus. The reason may be that the output of each convolutional layer is used as part of the final embedding, which reduces the risk of node over-smooth and improves the robustness of the model. Therefore, for the HM dataset, we set $\:l$ to be 3, and for the DB dataset, set $\:l$ be 2.

**Fig. 4:**
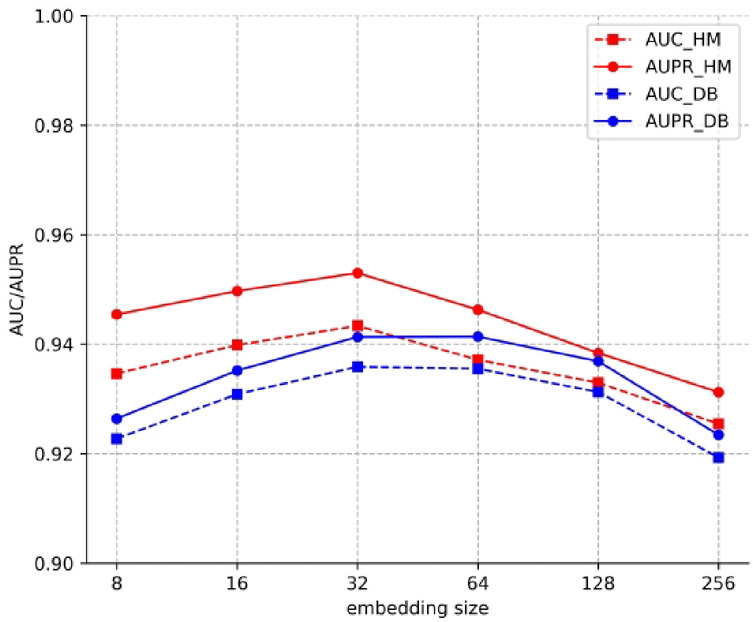
Performance comparison of different embedding size.

Third, we set *d* = 8, 16, 32, 64, 128, 256 in this experiment. The results are shown in Fig. 4. For dataset HM, the best results were obtained with the *d* is 32, and for the dataset DB, the best results were obtained with the *d* is 64.

### Performance of classifiers

For a machine learning problem, data and features determine the upper limit of machine learning, and models and algorithms only approach this upper limit. In this section, we investigate the effect of machine learning-based classifiers (MLC) on the performance of microbe-disease prediction. Three boosting algorithms, extreme gradient boosting (XGBoost), gradient boosting decision tree (GBDT) and adaptive boosting (Adaboost), two bagging algorithm, extremely randomized trees (ET) and random forest (RF), and two classic machine learning algorithms SVM and logistic regression (LR), are used to predict the association between microbes and diseases.

**Fig. 5:**
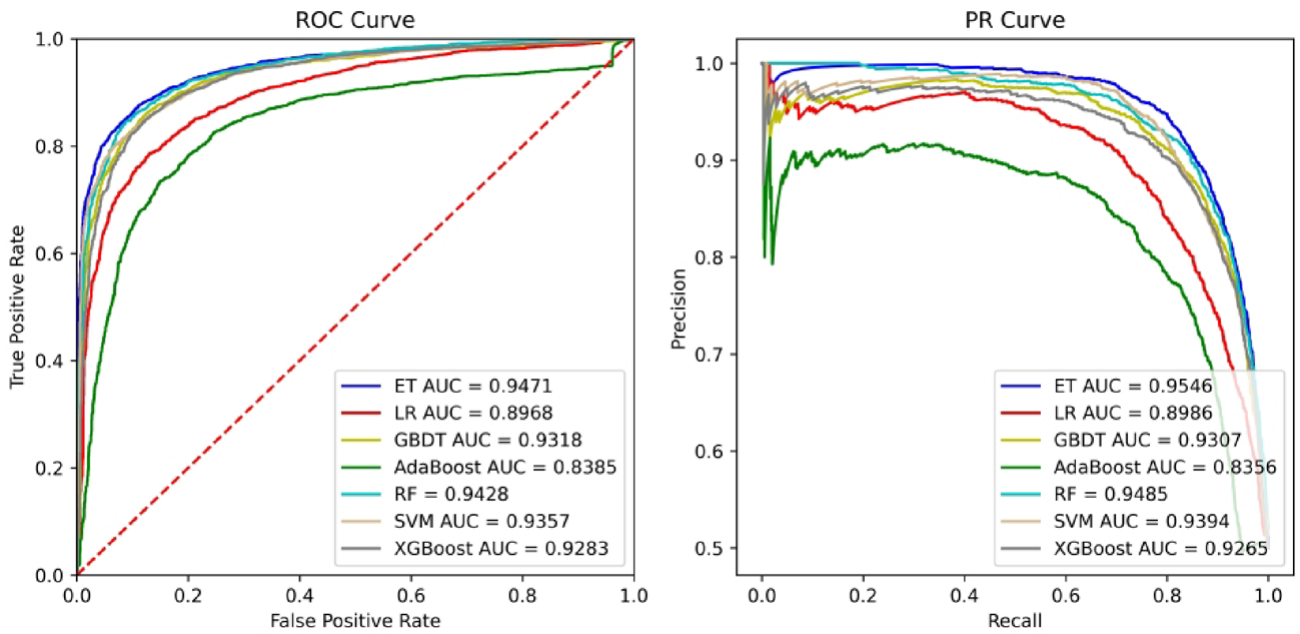
Performance comparison of different classifiers onHM.

**Fig. 6:**
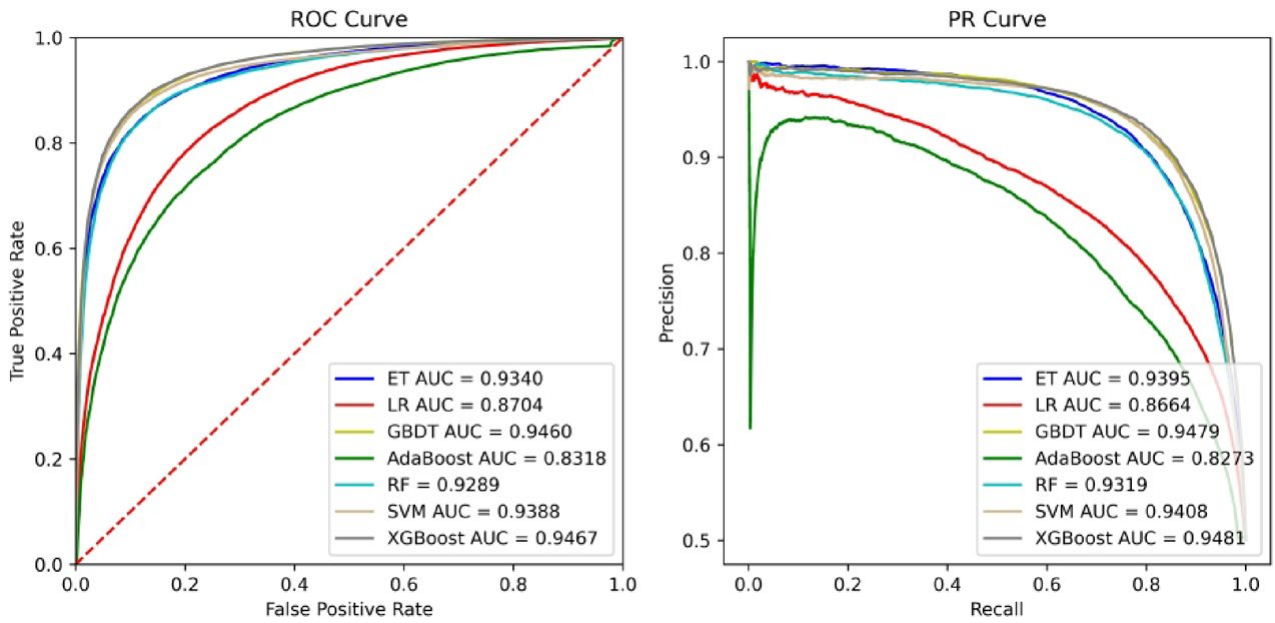
Performance comparison of different classifiers on DB.

Based on the same parameter setting, we trained seven independent classifiers to predict the association between microbes and diseases. The comparison results are shown in Figs. 5 and 6. It can be seen from the experimental results that the ET algorithm performs better on HM, and the XGBoost algorithm performs better on DB.

The experimental results in Figs. 5 and 6 show that the ET and XGBoost classifiers consistently outperform other methods on the HM and DB datasets, respectively. We selected ET as the primary classifier for our final model due to its robustness and efficiency. ET, a bagging-based ensemble method, builds multiple decision trees from the training data and averages their predictions. Unlike boosting algorithms (e.g., XGBoost, GBDT), which build trees sequentially to correct the errors of prior trees, ET introduces a higher level of randomness by selecting random split points for features. This increased randomness often helps to reduce model variance and prevent overfitting, which is particularly beneficial when dealing with potentially noisy biological data and high-dimensional embedding features.

While boosting methods can sometimes achieve slightly higher performance by aggressively minimizing bias, they can be more sensitive to hyperparameter tuning and noise. Given that ET provided highly competitive and stable performance across both benchmark datasets, we concluded it offered the best balance of accuracy, robustness, and generalizability for the microbe-disease prediction task.

### Ablation study

To rigorously assess the contribution of each component within our proposed FGCNMF pipeline, we conducted an ablation study. The primary goal was to disentangle the performance gains attributable to our novel embedding method from the effects of the downstream classifier. To achieve this, we compared our final FGCNMF embeddings against seven other feature representation methods: LINE, SDNE, GraRep, DeepWalk, Node2Vec, HOPE and Matrix Factorization only (MF-only).

We generated embeddings for all nodes in the HM and DB datasets using these eight methods. Subsequently, we used these embeddings as input features to train two top-performing classifiers from our earlier analysis: ET and XGBoost. By keeping the classifiers constant and varying the embedding source, we can directly attribute performance differences to the quality of the embeddings themselves.

The results of this ablation study, presented in Table 2, clearly demonstrate the superiority of our complete FGCNMF embedding framework. Across both datasets and for both classifiers, the FGCNMF embeddings consistently yielded the highest AUC and AUPR scores. This confirms that the primary driver of our model’s state-of-the-art performance is the high-quality feature representation learned by the FGCNMF pipeline, rather than a coincidental synergy with a single classifier.

**Table 2:** Ablation study results comparing different embedding methods on the HM and DB datasets using ET and XGBoost classifiers.

Data	Embedding model	Classifier	AUC	AUPR
HM	FGCNMF (Ours)	ET	**0.9471 ± 0.0075**	**0.9546 ± 0.0077**
		XGBoost	0.9287 ± 0.0157	0.9306 ± 0.0159
	LINE	ET	0.9110 ± 0.0154	0.9207 ± 0.0108
		XGBoost	0.9061 ± 0.0219	0.9053 ± 0.0214
	SDNE	ET	0.7884 ± 0.0609	0.7770 ± 0.0344
		XGBoost	0.8279 ± 0.0359	0.8488 ± 0.0325
	GraRep	ET	0.8790 ± 0.0335	0.8746 ± 0.0415
		XGBoost	0.8723 ± 0.0214	0.8709 ± 0.0190
	DeepWalk	ET	0.9399 ± 0.0226	0.9499 ± 0.0149
		XGBoost	0.9210 ± 0.0178	0.9314 ± 0.0139
	Node2Vec	ET	0.8287 ± 0.0297	0.8401 ± 0.0280
		XGBoost	0.8402 ± 0.0235	0.8544 ± 0.0215
	HOPE	ET	0.9279 ± 0.0103	0.9306 ± 0.0069
		XGBoost	0.9117 ± 0.0218	0.9059 ± 0.0291
	MF-only	ET	0.9215 ± 0.0094	0.9188 ± 0.0102
		XGBoost	0.9034 ± 0.0110	0.8714 ± 0.0160
DB	FGCNMF (Ours)	ET	0.9340 ± 0.0040	0.9395 ± 0.0060
		XGBoost	**0.9467 ± 0.0033**	**0.9481 ± 0.0034**
	LINE	ET	0.8740 ± 0.0069	0.8748 ± 0.0091
		XGBoost	0.9098 ± 0.0066	0.9108 ± 0.0080
	SDNE	ET	0.7369 ± 0.0119	0.7206 ± 0.0139
		XGBoost	0.7833 ± 0.0083	0.7893 ± 0.0105
	GraRep	ET	0.8185 ± 0.0115	0.8008 ± 0.0151
		XGBoost	0.8638 ± 0.0090	0.8560 ± 0.0116
	DeepWalk	ET	0.9446 ± 0.0038	0.9465 ± 0.0040
		XGBoost	0.9456 ± 0.0037	0.9477 ± 0.0044
	Node2Vec	ET	0.7985 ± 0.0113	0.7778 ± 0.0134
		XGBoost	0.8361 ± 0.0097	0.8316 ± 0.0109
	HOPE	ET	0.9339 ± 0.0042	0.9345 ± 0.0054
		XGBoost	0.9309 ± 0.0047	0.9225 ± 0.0057
	MF-only	ET	0.9103 ± 0.0051	0.9087 ± 0.0059
		XGBoost	0.9248 ± 0.0046	0.9211 ± 0.0048

The best-performing results are in bold.

### Computational efficiency analysis

A core design goal of FGCNMF was to achieve high predictive accuracy with exceptional computational efficiency. To substantiate the “Fast” claim in our model’s name, we provide both a theoretical complexity analysis and a comprehensive empirical runtime comparison.

FGCNMF’s efficiency stems from its two-stage, non-iterative design. The use of Randomized SVD for matrix factorization has a complexity of approximately O(*N*×*d*^2^+|*E*|)), where *N* is the number of nodes, *d* is the embedding dimension and |*E*| is the number of edges. This is a highly efficient one-shot calculation compared to iterative methods. The enhancement step involves *l* sparse-dense matrix multiplications, with a complexity of O(*l*×|*E*|×*d*). This is a lightweight, parameter-free operation.

To provide practical evidence of this efficiency, we conducted a runtime comparison on the large DB dataset. We measured the total time required to complete a single fold of the 5-fold cross-validation process. For methods requiring a separate classifier (e.g., classic embedding methods), this time includes both embedding generation and XGBoost training. For end-to-end GCNs, this is the total training time for a fixed 100 epochs. All experiments were performed on the same hardware with the following specifications: an Intel(R) Xeon(R) Gold 5418Y CPU @ 3.80 GHz, 512 GB of RAM, and an NVIDIA L40 GPU.

The results in Table 3 are conclusive. FGCNMF is dramatically faster than all other evaluated methods. It completes the task in just over one second. This represents a 2x to 8x speedup over classic graph embedding techniques and an even more significant 50x to 60x speedup compared to modern, complex GCN architectures. This outstanding computational efficiency, combined with its state-of-the-art predictive performance, firmly establishes FGCNMF as a powerful and highly practical tool for large-scale microbe-disease association prediction.

**Table 3:** Comparison of training time for a single cross-validation fold on the DB dataset.

Model	Training time(s)
FGCNMF (Ours)	**1.13**
LINE + Xgboost	4.41
SDNE + Xgboost	2.29
HOPE + Xgboost	3.88
DeepWalk + Xgboost	2.94
Node2Vec + Xgboost	8.76
GraRep + Xgboost	2.71
GAT	70.39
GraphSAGE	60.34
GraphTransformer	71.07
HGCLWithDiffusion	63.38

The best-performing results are in bold.

### Performance comparison with LightGCN

LightGCN^29^ is a widely used, simplified graph neural network that excels at learning node embeddings for recommendation tasks. It is conceptually similar to our approach in that it uses graph convolutions to refine representations. To provide a direct and meaningful comparison, we constructed two baselines based on it:

1. LightGCN: This refers to the standard, end-to-end LightGCN model trained with its native Bayesian Personalized Ranking (BPR) loss function to directly output a prediction score for a given node pair.
2. LightGCN + MLC: This baseline was designed to mirror our FGCNMF pipeline to provide direct comparison of the embedding quality. First, we used a trained LightGCN model to generate final node embeddings. Then, these static embeddings were used as input features for a downstream classifier. Here, MLC stands for Machine Learning-based Classifier, and for this baseline, we used the same Extra-Trees classifier employed in our FGCNMF model.

**Fig. 7:**
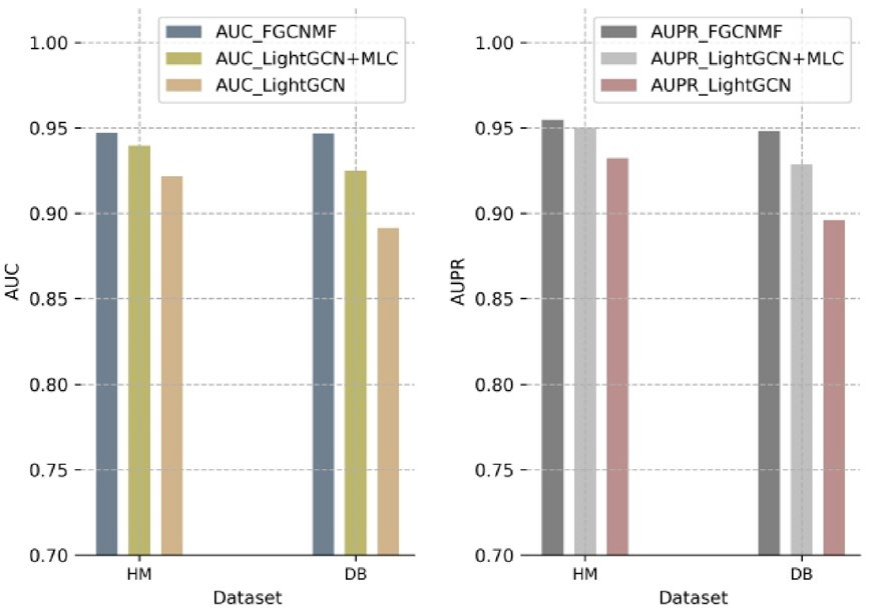
Performance comparison with LightGCN.

This comparison allows us to differentiate the performance of our fast, non-iterative embedding strategy (FGCNMF) from a standard, iteratively trained GCN embedding strategy (LightGCN + MLC), while also comparing against an end-to-end GCN framework (LightGCN). The results of the 5-fold cross-validation are shown in Fig. 7. The data clearly demonstrates that our FGCNMF model outperforms both LightGCN-based approaches, highlighting the effectiveness of our specialized feature extraction and prediction pipeline.

### Performance comparison with advanced GCN models

To rigorously contextualize FGCNMF’s performance within the contemporary landscape of graph learning, we benchmarked it against several state-of-the-art GCN models. The selected baselines represent key advancements in the field:

1. GraphSAGE: A prominent inductive GCN framework that aggregates features from a node’s local neighborhood.
2. GraphTransformer: A powerful architecture employing self-attention mechanisms, allowing nodes to weigh the importance of all other nodes in the graph.
3. HGCLWithDiffusion: A cutting-edge model that integrates heterogeneous graph contrastive learning with graph diffusion to learn robust representations.

Based on the torch_geometric(v 2.7.0) library, we implemented and optimized each baseline for the microbe-disease prediction task. The comparative results under the 5-fold cross-validation setting are presented in Table 4.

**Table 4:** Performance comparison with advanced GCN models.

Data	GCN Model	AUC	AUPR
HM	FGCNMF(Ours)	0.9471 ± 0.0075	0.9546 ± 0.0077
	GraphSAGE	**0.9449 ± 0.0124**	0.9514 ± 0.0098
	GraphTransformer	0.9535 ± 0.0067	**0.9573 ± 0.0062**
	HGCLWithDiffusion	0.8910 ± 0.0192	0.9093 ± 0.0132
DB	FGCNMF(Ours)	**0.9467 ± 0.0033**	**0.9481 ± 0.0034**
	GraphSAGE	0.9073 ± 0.0066	0.9073 ± 0.0069
	GraphTransformer	0.9328 ± 0.0050	0.9296 ± 0.0057
	HGCLWithDiffusion	0.7975 ± 0.0114	0.8187 ± 0.0100

The best-performing results are in bold.

The results in Table 4 reveal several key insights. On the smaller HM dataset, the performance is highly competitive, with the GraphTransformer model achieving the best AUPR score, while our FGCNMF model is a very close second and outperforms GraphSAGE. However, on the larger and more challenging DB dataset, FGCNMF clearly establishes state-of-the-art performance, surpassing all advanced GCNs, including the GraphTransformer, by a significant margin in both AUC and AUPR.

Notably, the complex HGCLWithDiffusion model did not generalize well to this task, suggesting that its architecture may be highly specialized for other problem domains or requires larger-scale data. The outstanding performance of FGCNMF, particularly on the DB dataset, is significant. It demonstrates that our streamlined and efficient hybrid approach, combining fast matrix factorization for a global view with simple spatial convolutions for local refinement, is a more effective strategy for this task than more complex, parameter-heavy models. This highlights the value of FGCNMF as a practical framework that achieves an exceptional balance between predictive power and model simplicity.

### Performance comparison with baseline

The comparison results with the baseline method are shown in Table 5. We conduct FGCNMF for 30 times on 5-fold CV to reduce the impact caused by sample division, and the mean values of AUPR achieves 95.46% on HM, and 94.81% on DB, which are both obvious higher than those of the comparison methods.

**Table 5:** The 5-CV prediction performance of different models.

Dataset	Model	AUC	AUPR
HM	FGCNMF (Ours)	*0.9471* $\:\pm\:$ *0.0075*	**0.9546** $\:\pm\:$ **0.0077**
	GATMDA^24^	**0.9554** $\:\pm\:$ **0.0184**	*0.9334* $\:\pm\:$ *0.0417*
	BRWMDA^30^	0.8916$\:\pm\:$0.0029	0.9064$\:\pm\:$0.0152
	KATZHMDA^13^	0.8703$\:\pm\:$0.0199	0.8807$\:\pm\:$0.0167
	GRNMFHMDA^31^	0.8806$\:\pm\:$0.0156	0.8914$\:\pm\:$0.0162
DB	FGCNMF (Ours)	**0.9467** $\:\pm\:$ **0.0033**	**0.9481** $\:\pm\:$ **0.0034**
	GATMDA^24^	*0.9307* $\:\pm\:$ *0.0079*	*0.9211* $\:\pm\:$ *0.0088*
	BRWMDA^30^	0.8266$\:\pm\:$0.0031	0.8031$\:\pm\:$0.0041
	KATZHMDA^13^	0.6779$\:\pm\:$0.0141	0.6785$\:\pm\:$0.0163
	GRNMFHMDA^31^	0.8609$\:\pm\:$0.0047	0.8669$\:\pm\:$0.0060

The best and second-best performing results are highlighted in bold and italic, respectively.

A closer analysis of the results in Table 5 reveals important insights into the interplay between model architecture and dataset characteristics. On the smaller and denser HM dataset, GATMDA achieves a slightly higher AUC score than FGCNMF. This can be attributed to GATMDA’s core Graph Attention Network (GAT) mechanism, which learns to assign variable importance weights to neighboring nodes. In a dense graph like HM, where local neighborhoods can be complex, this attention mechanism is highly effective at identifying the most critical connections. Our model’s simpler, unweighted convolutions, while much faster, may not capture these fine-grained local importance signals as effectively.

However, it is crucial to note two points. First, on the same HM dataset, FGCNMF achieves a significantly higher AUPR (0.9546 vs. 0.9334), a metric often more relevant for imbalanced biological data, suggesting superior performance in identifying true positive associations. Second, on the larger and sparser DB dataset, FGCNMF’s efficient and robust architecture demonstrates its strength, outperforming all baselines, including GATMDA, on both AUC and AUPR. This suggests that our approach scales more effectively and is better suited for larger, more challenging networks.

This analysis provides a clear avenue for future improvement, integrating a lightweight or simplified attention mechanism into the FGCNMF framework could potentially combine the strengths of both approaches, leading to a model with enhanced performance on dense networks while retaining the speed and scalability that make it so effective on larger ones.

### Performance on external dataset

To verify the generalizability of the FGCNMF architecture, we applied our entire prediction pipeline to three external microbe-drug association datasets (see Table 6). This involves constructing the network, generating embeddings, and training a classifier from scratch for each new dataset. For these validation experiments, we consistently used the Extra-Trees (ET) algorithm as the downstream classifier. This methodological choice is crucial for a fair evaluation. Our analysis in Section "Performance of Classifiers" showed that while boosting models like XGBoost can achieve peak performance on a specific dataset (e.g., DB), the ET classifier provided highly competitive and stable performance across both of our primary benchmarks. By fixing the classifier to a robust and high-performing option like ET, we ensure that the results on these new datasets are a direct reflection of the FGCNMF embedding framework’s ability to generate powerful predictive features, rather than the outcome of dataset-specific classifier tuning.

**Table 6:** The statistical information of microbe-drug datasets.

Dataset	Microbe	Drug	Links	Sparseness
MDAD	173	1373	2470	0.0104
aBiofilm	140	1720	2884	0.0120
DrugVirus	95	175	933	0.0561

In this experiment, the depth of the convolutional layer is set to 2, and the classifier adopts the ET algorithm. Referring to the number of samples in the dataset, the embedding size is set to 64 for the first two datasets and 32 for the last dataset. Table 7 shows the results of FGCNMF and other baselines on these three datasets. Overall, FGCNMF achieves SOTA performance on the first two datasets. The area under the precision-recall curve (AUPR) of FGCNMF have reached to 97.42% and 97.86%, respectively, which are significantly superior to the previous method. The AUC and AUPR of the third dataset are 89.04% and 88.82%, which are 0.82% and 1.56% lower than the optimal GCNMDA^14^, respectively.

**Table 7:** The summary of model performance on microbe-drug datasets.

Dataset	Model	AUC	AUPR
MDAD	FGCNMF (Ours)	**0.9667** $\:\pm\:$ **0.0025**	**0.9742** $\:\pm\:$ **0.0017**
	GCNMDA^14^	*0.9423* $\:\pm\:$ *0.0105*	*0.9376* $\:\pm\:$ *0.0114*
	IMCMDA^32^	0.7466$\:\pm\:$0.0102	0.7773$\:\pm\:$0.0113
	WNN_GIP^33^	0.8721$\:\pm\:$0.0162	0.8922$\:\pm\:$0.0137
	GCMDR^34^	0.8485$\:\pm\:$0.0062	0.8509$\:\pm\:$0.0040
aBiofilm	FGCNMF (Ours)	**0.9715** $\:\pm\:$ **0.0052**	**0.9786** $\:\pm\:$ **0.0025**
	GCNMDA^14^	*0.9517* $\:\pm\:$ *0.0033*	*0.9488* $\:\pm\:$ *0.0031*
	IMCMDA^32^	0.7750$\:\pm\:$0.0096	0.8572$\:\pm\:$0.0049
	WNN_GIP^33^	0.9019$\:\pm\:$0.0187	0.9408$\:\pm\:$0.0132
	GCMDR^34^	0.8772$\:\pm\:$0.0076	0.8847$\:\pm\:$0.0061
DrugVirus	FGCNMF (Ours)	*0.8904* $\:\pm\:$ *0.0086*	*0.8882* $\:\pm\:$ *0.0098*
	GCNMDA^14^	**0.8986** $\:\pm\:$ **0.0305**	**0.9038** $\:\pm\:$ **0.0372**
	IMCMDA^32^	0.6235$\:\pm\:$0.0245	0.6962$\:\pm\:$0.0302
	WNN_GIP^33^	0.8002$\:\pm\:$0.0192	0.8436$\:\pm\:$0.0183
	GCMDR^34^	0.8243$\:\pm\:$0.0168	0.8206$\:\pm\:$0.0141

The best and second-best performing results are highlighted in bold and italic, respectively.

### Case studies for novel prediction validation

To demonstrate the practical utility and hypothesis-generation capabilities of FGCNMF, we conducted case studies to assess its ability to predict novel, scientifically plausible associations for two well-known diseases: asthma and inflammatory bowel disease (IBD). This analysis was designed to simulate a real-world discovery scenario where the goal is to identify new research targets. The methodology was as follows:

1. The FGCNMF model was first trained on the complete HM dataset, leveraging all 450 known microbe-disease associations to learn a comprehensive model of these relationships.
2. The trained model was then used to predict association scores for both asthma and IBD against every microbe in the dataset.
3. For each disease, the predicted microbes were ranked by their association scores. Crucially, we then filtered out any associations that were already present in our original training set of 450 known links. This left us with a ranked list of purely novel predictions.
4. We selected the top 20 novel candidate microbes for each disease and performed a comprehensive literature search to determine if these data-driven hypotheses were supported by independent biological or clinical evidence not captured in our original dataset.

Asthma is a heterogeneous inflammatory disease with a well-established, albeit complex, link to the microbiome^35^. Our model successfully generated a list of high-confidence novel predictions. Upon validating the top 20 candidates (as detailed in Table 8), our literature review confirmed that 16 of them have been independently reported in scientific publications as being associated with asthma. This 80% confirmation rate for novel predictions strongly underscores our model’s ability to act as an effective hypothesis generation engine, capable of identifying clinically relevant microbes that are missing from existing curated databases.

Similarly, IBD serves as a quintessential example of a condition driven by gut microbiome dysbiosis^36^, making it an ideal test case^37^. When applied to IBD, FGCNMF’s ability to generate valid novel hypotheses was even more pronounced. After validating the top 20 predicted novel microbes (Table 9), we found that 17 of them, representing an 85% confirmation rate, are indeed recognized in published research as being associated with IBD. This result powerfully demonstrates the robustness of FGCNMF and solidifies its potential as a valuable computational tool for prioritizing novel targets in microbiome-centric disease research.

**Table 8:** The top 20 asthma-related candidate microbes.

Rank	Microbe	Evidence	Rank	Microbe	Evidence
1	Clostridium difficile	PMID:25,974,301	11	Firmicutes	PMID:23,265,859
2	Burkholderia	PMID:24,451,910	12	Fusobacterium	PMID: 28,486,933
3	Actinobacteria	PMID:23,265,859	13	Escherichia coli	PMID:29,161,804
4	Lactobacillus	PMID:20,592,920	14	Dietzia maris	Unconfirmed
5	Propionibacterium acnes	PMID: 30,185,226	15	Pseudomonas	PMID:27,076,584
6	Enterococcus	PMID:29,788,027	16	Clostridium leptum	PMID:29,445,257
7	Staphylococcus epidermidis	PMID: 29,569,134	17	Streptococcus	PMID:17,950,502
8	Propionibacterium	PMID: 13,268,970	18	Verrucomicrobiaceae	Unconfirmed
9	Staphylococcus aureus	PMID:12,743,582	19	Bacteroidaceae	PMID:28,947,029
10	Stenotrophomonas maltophilia	Unconfirmed	20	Lysobacter	Unconfirmed

**Table 9:** The top 20 IBD-related candidate microbes.

Rank	Microbe	Evidence	Rank	Microbe	Evidence
1	Bacteroidetes	PMID:25,307,765	11	Corynebacterium	Unconfirmed
2	Firmicutes	PMID:25,307,765	12	Staphylococcus aureus	Unconfirmed
3	Staphylococcus	PMID: 27,239,107	13	Bacteroidaceae	PMID:17,897,884
4	Prevotella	PMID:25,307,765	14	Parabacteroides	PMID:25,307,765
5	Clostridium difficile	Unconfirmed	15	Alistipes	PMID:28,877,044
6	Porphyromonadaceae	PMID:29,573,237	16	Oxalobacter formigenes	PMID:15,610,315
7	Rikenellaceae	PMID: 31,708,890	17	Enterobacteriaceae	PMID:24,629,344
8	Clostridia	PMID: 31,142,855	18	Veillonella	PMID:28,842,640
9	Citrobacter	PMID: 30,342,282	19	Acinetobacter	PMID:22,720,094
10	Ruminococcaceae	PMID: 31,379,797	20	Clostridium coccoides	PMID:19,235,886

## Conclusion

In this study, we introduced FGCNMF, a new graph embedding framework that integrates fast matrix factorization with spatial graph convolutions to predict microbe-disease associations. By efficiently capturing both global network structure and local neighborhood information, FGCNMF achieves state-of-the-art performance on benchmark datasets, even outperforming more complex GCN architectures. The results underscore the effectiveness of our lightweight and practical approach for this important bioinformatics task.

However, we recognize that our model has certain limitations that offer clear directions for future improvement, particularly concerning its scalability and adaptability.

One of the primary limitations of the current FGCNMF framework is its transductive nature. The model learns specific embeddings for the nodes present in the training graph. If a new microbe or disease is discovered, its inclusion requires rebuilding the entire heterogeneous network, recalculating the similarity matrix, and retraining the entire model from scratch. This process can be computationally expensive and limits the model’s utility in dynamic, real-world scenarios where biological databases are continuously updated.

To address this challenge, future work will focus on extending FGCNMF to an inductive framework capable of handling new entities efficiently. We propose two primary strategies:

1. Inductive inference for new nodes. A semi-inductive approach can be developed to accommodate new nodes without full retraining. When a new microbe *m_new* is introduced, we can first calculate its similarity to all existing microbes in the database. Its initial embedding, *F(0)_new*, can then be approximated as a weighted average of the embeddings of its k most similar neighbors:

$$\:F\left(0\right)\_new\:=\:{\sum\:}_{i}\left(sim\right(m\_new,m\_i)\times\:\:F(0)\_i)/{\sum\:}_{i}sim(m\_new,m\_i)$$

where $\:m\_i$ are the known neighbors and *sim()* is the similarity function. Following this initialization, a few steps of localized graph convolution can be applied to refine this embedding based on its new connections, without updating the embeddings of the rest of the graph. This would offer a fast and efficient method for generating predictions for new entities.

(2) Transition to a fully inductive framework. A more powerful long-term solution is to redesign the model to be fully inductive from the start. This would involve incorporating rich initial node features beyond graph structure, such as genomic sequences or functional annotations for microbes and clinical symptom profiles or gene expression data for diseases. By training a GCN (like GraphSAGE or GAT) to learn a function that maps these features to the final embedding space, the model would be inherently capable of generating embeddings for entirely unseen nodes, thus providing true generalization and eliminating the need for retraining.

We believe that pursuing these directions will significantly enhance the practical applicability of our work. There is still ample room for improvement in microbe-disease association prediction, and adapting powerful graph representation learning algorithms for dynamic and evolving biological data remains a critical and exciting challenge.

## Data Availability

The source code and dataset are available from the corresponding author by request.

## References

[1] Ma, W., Huang, C., Zhou, Y., Li, J. & Cui, Q. MicroPattern: A web-based tool for microbe set enrichment analysis and disease similarity calculation based on a list of microbes. *Sci. Rep.* **7**, 40200. 10.1038/srep40200 (2017). 28071710 10.1038/srep40200 PMC5223220

[2] Lewis, D. J., Chan, W. H., Hinojosa, T., Hsu, S. & Feldman, S. R. Mechanisms of microbial pathogenesis and the role of the skin Microbiome in psoriasis: A review. *Clin. Dermatol.* **37**, 160–166. 10.1016/j.clindermatol.2019.01.011 (2019). 30981296 10.1016/j.clindermatol.2019.01.011

[3] Dréno, B. et al. Microbiome in healthy skin, update for dermatologists. *J. Eur. Acad. Dermatol. Venereol.* **30**, 2038–2047. 10.1111/jdv.13965 (2016). 27735094 10.1111/jdv.13965 PMC6084363

[4] Ihekweazu, F. D. & Versalovic, J. Development of the pediatric gut microbiome: Impact on health and disease. *Am. J. Med. Sci.* **356**, 413–423. 10.1016/j.amjms.2018.08.005 (2018). 30384950 10.1016/j.amjms.2018.08.005 PMC6268214

[5] Sata, Y., Marques, F. Z. & Kaye, D. M. The emerging role of gut dysbiosis in cardio-metabolic risk factors for heart failure. *Curr. Hypertens. Rep.* **22**, 38. 10.1007/s11906-020-01046-0 (2020). 32385705 10.1007/s11906-020-01046-0

[6] Hiippala, K. et al. Novel* Odoribacter splanchnicus* strain and its outer membrane vesicles exert immunoregulatory effects in vitro. *Front. Microbiol.* **11**, 575455. 10.3389/fmicb.2020.575455 (2020). 33281770 10.3389/fmicb.2020.575455 PMC7689251

[7] Cong, J. & Zhang, X. How human microbiome talks to health and disease. *Eur. J. Clin. Microbiol. Infect. Dis.* **37**, 1595–1601. 10.1007/s10096-018-3263-1 (2018). 29682676 10.1007/s10096-018-3263-1

[8] Zhao, Y., Wang, C. C. & Chen, X. Microbes and complex diseases: from experimental results to computational models. *Brief. Bioinform*. 10.1093/bib/bbaa158 (2021). 10.1093/bib/bbaa158 32766753

[9] Bao, W., Jiang, Z. & Huang, D. S. Novel human microbe-disease association prediction using network consistency projection. *BMC Bioinform.* **18**, 543. 10.1186/s12859-017-1968-2 (2017). 10.1186/s12859-017-1968-2 PMC5751545 29297304

[10] Yan, C., Duan, G., Wu, F. X., Pan, Y. & Wang, J. M. C. H. M. D. A. Predicting microbe-disease associations based on similarities and low-rank matrix completion. *IEEE/ACM Trans. Comput. Biol. Bioinform*. **18**, 611–620. 10.1109/tcbb.2019.2926716 (2021). 31295117 10.1109/TCBB.2019.2926716

[11] Yi, H. C. et al. Learning representations to predict intermolecular interactions on large-scale heterogeneous molecular association network. *iScience* 23, 101261, (2020). 10.1016/j.isci.2020.101261 10.1016/j.isci.2020.101261 PMC7317230 32580123

[12] Guo, Z. H. et al. A learning based framework for diverse biomolecule relationship prediction in molecular association network. *Commun. Biol.* **3**, 118. 10.1038/s42003-020-0858-8 (2020). 32170157 10.1038/s42003-020-0858-8 PMC7070057

[13] Chen, X., Huang, Y. A., You, Z. H., Yan, G. Y. & Wang, X. S. A novel approach based on KATZ measure to predict associations of human microbiota with non-infectious diseases. *Bioinformatics* **33**, 733–739. 10.1093/bioinformatics/btw715 (2017). 28025197 10.1093/bioinformatics/btw715

[14] Long, Y., Wu, M., Kwoh, C. K., Luo, J. & Li, X. Predicting human microbe-drug associations via graph convolutional network with conditional random field. *Bioinf. (Oxford England)*. **36**, 4918–4927. 10.1093/bioinformatics/btaa598 (2020). 10.1093/bioinformatics/btaa598 PMC7559035 32597948

[15] Long, Y. & Luo, J. WMGHMDA: A novel weighted meta-graph-based model for predicting human microbe-disease association on heterogeneous information network. *BMC Bioinform.* **20**, 541. 10.1186/s12859-019-3066-0 (2019). 10.1186/s12859-019-3066-0 PMC6824056 31675979

[16] Fan, Y., Chen, M., Zhu, Q. & Wang, W. Inferring disease-associated microbes based on multi-data integration and network consistency projection. *Front. Bioeng. Biotechnol.* **8**, 831. 10.3389/fbioe.2020.00831 (2020). 32850711 10.3389/fbioe.2020.00831 PMC7418576

[17] Yin, M. M., Liu, J. X., Gao, Y. L., Kong, X. Z. & Zheng, C. H. NCPLP: A novel approach for predicting microbe-associated diseases with network consistency projection and label propagation. *IEEE Trans. Cybern Pp*. 10.1109/tcyb.2020.3026652 (2020). 10.1109/TCYB.2020.3026652 33119529

[18] Wang, F. et al. Laplacian regularized least squares for human microbe-disease association prediction. *Sci. Rep.* **7**, 7601. 10.1038/s41598-017-08127-2 (2017). 28790448 10.1038/s41598-017-08127-2 PMC5548838

[19] Peng, L. H., Yin, J., Zhou, L., Liu, M. X. & Zhao, Y. Human microbe-disease association prediction based on adaptive boosting. *Front. Microbiol.* **9**, 2440. 10.3389/fmicb.2018.02440 (2018). 30356751 10.3389/fmicb.2018.02440 PMC6189371

[20] Li, H. et al. Identifying microbe-disease association based on a novel back-propagation neural network model. *IEEE/ACM Trans. Comput. Biol. Bioinform*. 10.1109/tcbb.2020.2986459 (2020). 32305935 10.1109/TCBB.2020.2986459

[21] Liu, Y. et al. Prediction of microbe-disease associations based on deep matrix factorization using bayesian personalized ranking. *IEEE/ACM Trans. Comput. Biol. Bioinform*. **18**, 1763–1772. 10.1109/tcbb.2020.3018138 (2021). 32816678 10.1109/TCBB.2020.3018138

[22] Peng, L., Shen, L., Liao, L., Liu, G. & Zhou, L. R. N. M. F. M. D. A. A microbe-disease association identification method based on reliable negative sample selection and logistic matrix factorization with neighborhood regularization. *Front. Microbiol.* **11**, 592430. 10.3389/fmicb.2020.592430 (2020). 33193260 10.3389/fmicb.2020.592430 PMC7652725

[23] Wang, G. et al. Heterogeneous graph contrastive learning with graph diffusion for drug repositioning. *J. Chem. Inf. Model.* **65**, 5771–5784. 10.1021/acs.jcim.5c00435 (2025). 40377926 10.1021/acs.jcim.5c00435

[24] Long, Y., Luo, J., Zhang, Y. & Xia, Y. Predicting human microbe-disease associations via graph attention networks with inductive matrix completion. *Brief. Bioinform*. 10.1093/bib/bbaa146 (2021). 10.1093/bib/bbaa146 32725163

[25] Ma, W. et al. An analysis of human microbe-disease associations. *Brief. Bioinform*. **18**, 85–97. 10.1093/bib/bbw005 (2017). 26883326 10.1093/bib/bbw005

[26] Janssens, Y. et al. Disbiome database: Linking the microbiome to disease. *BMC Microbiol.*10.1186/s12866-018-1197-5 (2018). 10.1186/s12866-018-1197-5 PMC5987391 29866037

[27] Kamneva, O. K. Genome composition and phylogeny of microbes predict their co-occurrence in the environment. *PLoS Comput. Biol.* **13**, e1005366. 10.1371/journal.pcbi.1005366 (2017). 28152007 10.1371/journal.pcbi.1005366 PMC5313232

[28] Zhang, J., Dong, Y., Wang, Y., Tang, J. & Ding, M. In *Twenty-Eighth International Joint Conference on Artificial Intelligence IJCAI-19* (2019).

[29] He, X. et al. Association for computing machinery, virtual event, China. In *Proceedings of the 43rd International ACM SIGIR Conference on Research and Development in Information Retrieval* 639–648 (2020).

[30] Yan, C., Duan, G., Wu, F. X., Pan, Y. & Wang, J. BRWMDA :Predicting microbe-Disease associations based on similarities and Bi-Random walk on disease and microbe networks. *IEEE/ACM Trans. Comput. Biol. Bioinform*. **17**, 1595–1604. 10.1109/tcbb.2019.2907626 (2020). 30932846 10.1109/TCBB.2019.2907626

[31] He, B. S., Peng, L. H. & Li, Z. Human microbe-disease association prediction with graph regularized non-negative matrix factorization. *Front. Microbiol.* **9**, 2560. 10.3389/fmicb.2018.02560 (2018). 30443240 10.3389/fmicb.2018.02560 PMC6223245

[32] Chen, X., Wang, L., Qu, J., Guan, N. N. & Li, J. Q. Predicting miRNA-disease association based on inductive matrix completion. *Bioinf.*. **34**, 4256–4265. 10.1093/bioinformatics/bty503 (2018). 10.1093/bioinformatics/bty503 29939227

[33] van Laarhoven, T. & Marchiori, E. Predicting drug-target interactions for new drug compounds using a weighted nearest neighbor profile. *PloS One*. **8**, e66952. 10.1371/journal.pone.0066952 (2013). 23840562 10.1371/journal.pone.0066952 PMC3694117

[34] Huang, Y. A., Hu, P., Chan, K. C. C. & You, Z. H. Graph convolution for predicting associations between MiRNA and drug resistance. *Bioinf.*. **36**, 851–858. 10.1093/bioinformatics/btz621 (2020). 10.1093/bioinformatics/btz621 31397851

[35] Fu, X. et al. Derived habitats of indoor microbes are associated with asthma symptoms in Chinese university dormitories. *Environ. Res.*10.1016/j.envres.2020.110501 (2020). 10.1016/j.envres.2020.110501 33221308

[36] Li, K. et al. Bacteroides Thetaiotaomicron relieves colon inflammation by activating Aryl hydrocarbon receptor and modulating CD4(+)T cell homeostasis. *Int. Immunopharmacol.*10.1016/j.intimp.2020.107183 (2020). 10.1016/j.intimp.2020.107183 33229197

[37] Lee, M. & Chang, E. B. Inflammatory bowel diseases (IBD) (Inflammatory bowel diseases and the microbiome: Uearching the crime scene for Clues). *Gastroenterology*10.1053/j.gastro.2020.09.056 (2020). 33253681 10.1053/j.gastro.2020.09.056 PMC8098834

